# Comparison of medial tibiofemoral joint mechanics between all-suture anchors and transtibial pullout technique for posterior medial meniscal root tears

**DOI:** 10.1186/s13018-023-04071-2

**Published:** 2023-08-09

**Authors:** Nadhaporn Saengpetch, Sutip Noowan, Artit Boonrod, Khananut Jaruwanneechai, Sermsak Sumanont, Chaiyanun Vijittrakarnrung

**Affiliations:** 1 Department of Orthopedics, Faculty of Medicine Ramathibodi Hospital, Mahidol University, 270, Rama VI Road, Ratchathewi District, Bangkok, 10400 Thailand; 2 Department of Orthopedics, Faculty of Medicine, Khon Kaen University, Khon kaen, 40002 Thailand

**Keywords:** Meniscus root, Root tear, Meniscus tear, Meniscus root repair, Transtibial pullout repair, All-suture anchor repair

## Abstract

**Background:**

The posterior medial meniscal root tear (PMMRT) seriously impacts the tibiofemoral joint biomechanics. Two available techniques for PMMRT repair include the transtibial pullout (TPO) repair and all-suture anchor (ASA) repair techniques. These techniques have not been compared biomechanically.

**Methods:**

A total of 20 fresh porcine cadaveric knee specimens were used. All 20 knees were randomly and evenly distributed into four groups (five specimens per group): (1) intact posterior meniscal root, (2) PMMRT, (3) TPO repair technique for PMMRT, and (4) ASA repair technique for PMMRT. The tibiofemoral contact mechanics were investigated using a pressure sensor. All knee specimens were tested by being loaded with 600 N axial compressive force at three different flexion angles (0°, 45°, and 90°). The contact surface area, contact pressure, peak pressure, and time-zero displacement were recorded.

**Results:**

The PMMRT caused a significant decrease in contact surface area, an increase in contact pressure, and peak pressure from the reference values observed in the intact meniscus group (*P* = 0.05, 0.016, and 0.008, respectively). After fixation, no significant difference was observed between the ASA and intact group. Meanwhile, significant differences were found between the TPO and intact group in terms of contact surface area, contact pressure, and peak pressure. In the comparison between the two techniques, the ASA group demonstrated higher contact surface area than the TPO group at the average knee flexion angle (*p* = 0.05).

**Conclusion:**

For most testing conditions, the ASA technique demonstrated superior biomechanical property in terms of contact surface area compared with the TPO technique under compressive loading conditions. The ASA technique could also restore the tibiofemoral contact mechanics to be comparable with those of the native intact knee. Meanwhile, a significant difference in tibiofemoral mechanics, compared with the intact knee, could be observed in the TPO technique.

## Introduction

The posterior medial meniscal root tear (PMMRT) is relatively common among middle-aged adults, with a reported incidence rate as high as 21.5% [1, 2]. Serving a crucial function, the meniscal root preserves hoop tension and prevents extrusion [3, 4]. Biomechanical studies demonstrated how the loading characteristic in the tibiofemoral joint is compromised after PMMRT, resulting in decreased contact area and increased contact pressure [5–7]. In a previous human cadaveric study, the consequence of PMMRT was indistinguishable from those of total medial meniscectomy in terms of peak tibiofemoral pressure, which potentially leads to the development and even acceleration of medial compartmental osteoarthritis [5]. Since conservative treatment of PMMRT showed unfavorable outcomes [8, 9], surgical repair of the PMMRT is currently the preferred option and has promising clinical outcomes in properly indicated patients [10–13].

Two common options—the transtibial pullout (TPO) repair and suture anchor repair techniques—can be used to reattach the PMMRT. The more popular technique is the TPO repair, which secures the meniscal root to its attachment using sutures that pass through the tibia tunnel and tie over the tibial cortex. This technique has been reported to obtain good tibiofemoral contact mechanics and improved functional outcomes [7, 13, 14]. Nevertheless, Feucht et al. [15] conducted a systematic review of the TPO method and observed a complete healing in only 62% of cases, based on magnetic resonance imaging (MRI) and second-look arthroscopy. LaPrade et al. [13] also reported a revision rate in 9.7% of patients who underwent the PMMRT repair with the TPO technique. The unsatisfactory results observed in these studies may be attributed, in part, to the suboptimal pullout strength exhibited by the employed repair techniques. Additionally, indirect refixation of the meniscus root through the use of TPO technique could potentially compromise the healing process which due to the decreased stiffness and increased micromotion experienced by the meniscus–suture complex under repetitive loads [16, 17]. These overall negative results highlight the need to improve the outcomes of PMMRT repair.

Proposed as an alternative option for PMMRT repair, the suture anchor technique has theoretical benefits over the TPO technique [16, 18], as it allows for direct refixation of the PMMRT at its tibial insertion site and eliminates the need for tunnel drilling. Kim et al. [19] reported a decreased incidence of incomplete healing after PMMRT when using the suture anchor technique compared to the TPO technique, suggesting that the anchor technique might be favored over the TPO technique. However, the current suture anchor technique still requires creating an accessory posteromedial portal, which could injure neurovascular structures [20]. Even with the aid of a specific curved passing guide, identifying the perfect direction to insert the suture anchor remains challenging [21]. Recently, Balke et al. have proposed a repair technique using an all-suture anchor (ASA) which use expanding intracortical sutures to fix the anchor, allowing for smaller drill holes with less bone disruption. Furthermore, this anchor insertion technique could be modified, enabling the surgeon to pull it into the bone instead of tapping it in through an additional posterior portal. They claimed that ASA technique could surpass all limitations of previous anchor suture repair techniques [22]. Previous studies have investigated the biomechanical outcomes of repair techniques for PMMRT [16, 19], but using the ASA technique for PMMRT repairs has not been biomechanically compared to any of other techniques.

The primary objectives of our study were to (1) compare the tibiofemoral contact mechanics between the TPO technique and ASA techniques for PMMRT repair via an in vitro porcine model and (2) assess the magnitude of the time-zero displacement of the posterior medial meniscal root in response to the maximal compression force of each testing condition. The hypothesis was that the ASA repair technique can provide superior biomechanical properties compared to the TPO technique and would restore the tibiofemoral contact mechanics to that of the native intact knee. In addition, we hypothesized that the amount of time-zero displacement would be lowest after the ASA repair technique.

## Materials and methods

### Specimen preparation

**Fig. 1:**
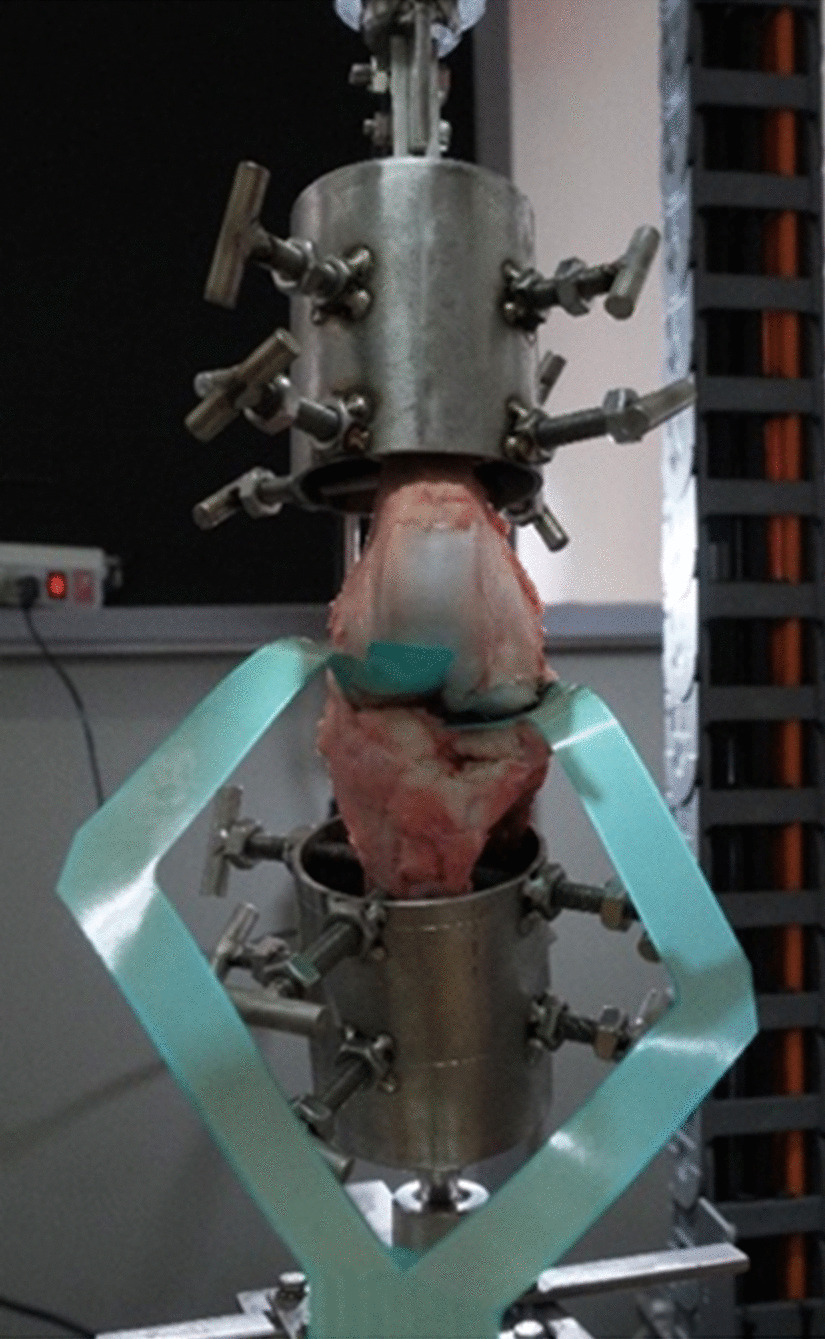
Biomechanical testing set up for 0° knee flexion. The depicted custom jig made it possible to control two axes of rotational and vertical motion during dynamic testing conditions. The femur and tibia were secured into the custom jig to simulate the motion of the knee joint. A capacitive pressure sensor (Tekscan model 4000) was inserted at the medial and lateral compartments

Twenty fresh porcine hindleg knee specimens with an average age range of 5–6 months were recruited and obtained from a local butcher. This sample size was determined by referencing the power analysis of prior analog biomechanical meniscal root [23, 24]. The day before preparation, the animals were exterminated. The porcine cadaveric model was selected due to its prior use in many previous biomechanical studies on meniscal repair [16, 25–27]. The current study was approved by the institutional research board committee (COA.MURA2021/868). For all specimens, the knee was dissected free of all extra-articular skin, subcutaneous tissue, muscles, and the patella. The fibula was also detached from the proximal tibia. The femur, tibia, cruciate ligaments, and collateral ligaments were preserved. The menisci were left attached to the tibial plateau. In addition, all specimens were scrutinized for the absence of femoral condyle hypoplasia, ligamentous injury, meniscal lesion, and cartilage degeneration. The femur, tibia, and fibula were cut 20 cm proximal and distal to the joint line. The femur and tibia were secured in a customized jig of a dynamic testing machine attached 10–12 cm from the joint line, with the tibial plateau aligned parallel to the bases (Fig. 1). All 20 knees were randomly and evenly distributed to one of four groups (with five specimens per group): (1) intact posterior meniscal root, (2) PMMRT, (3) TPO repair technique for PMMRT, and (4) ASA repair techniques for PMMRT. The posterior meniscal root attachments were clearly visualized from the posterior view of all knees. For every testing group except the intact group, sharp and complete radial cuts were made to the posterior medial meniscus roots with a scalpel at 5 mm medially from its attachment.

### Transtibial pullout repair technique

**Fig. 2:**
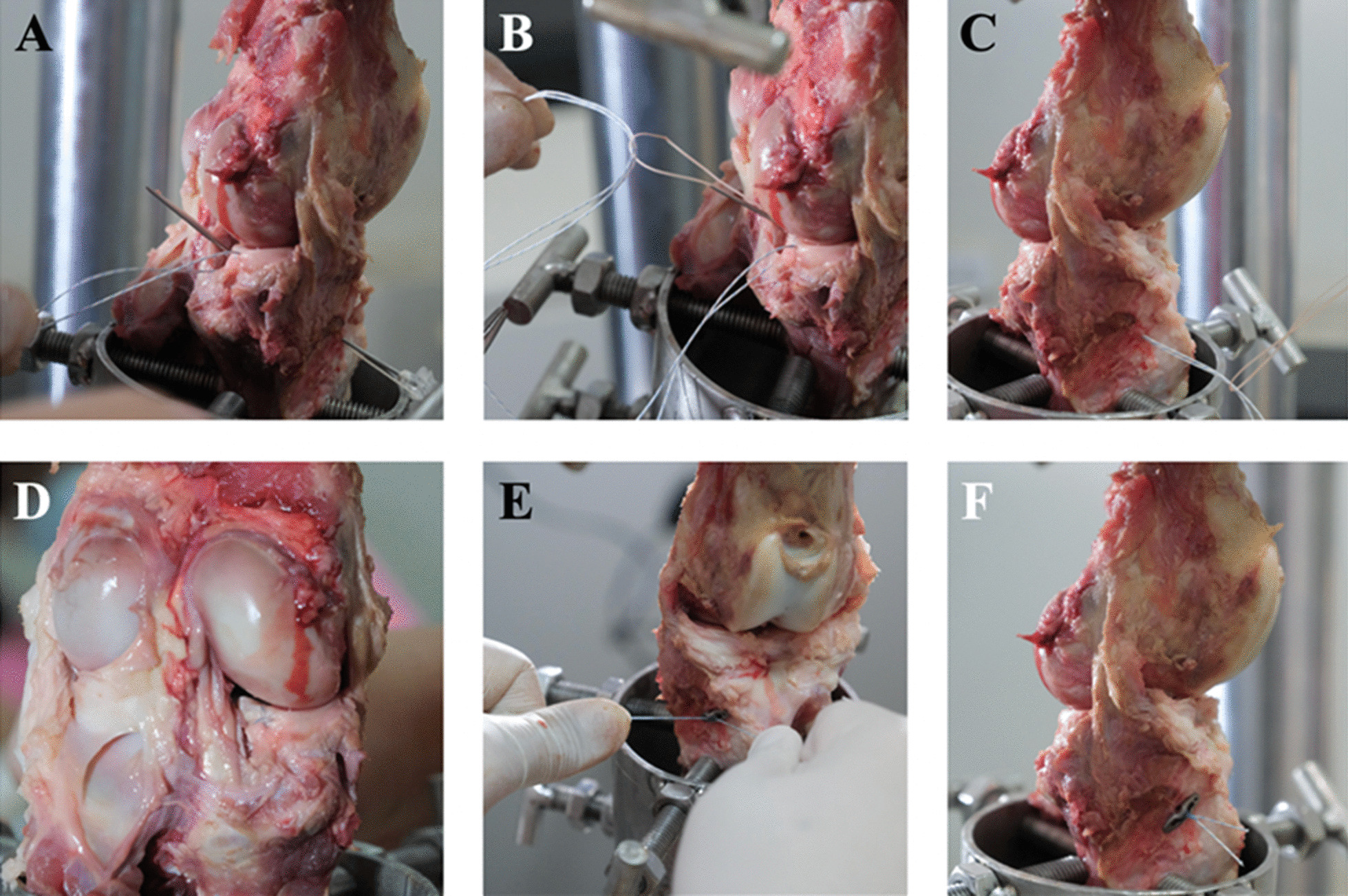
Steps for PMMRT repair with transtibial pullout repair technique. **A** After the meniscus was repaired using No. 2 Hi-Fi sutures via a modified Mason–Allen technique, the anterior cruciate ligament (ACL) tibial guide was used to create the tibial tunnel at the posterior medial meniscal root footprint, and a spinal needle was inserted. **B** Both ends of the Hi-Fi sutures were inserted through the looped suture. **C** Both ends of the Hi-Fi sutures were retrieved through the tibial tunnel at the anterior tibia. **D** View of the constructed suture from the posterior view of the knee. **E** Two ends of the Hi-Fi sutures were inserted into the free XO button and tied with five surgical knots. **F** The final transtibial pullout repair technique construct was illustrated

After the establishment of the PMMRT, the meniscal tissue was meticulously sutured utilizing the FirstPass Mini Suture Passer (Smith and Nephew, Andover, MA, USA), employing two non-absorbable number 2 Hi-Fi sutures (ConMed-Linvatec, Largo, FL, USA) in a modified Mason–Allen suture configuration, while allowing two suture ends to remain free. The passage of sutures was accomplished at a distance of 3 mm medially from the torn edge of the posterior horn of the medial meniscus in every specimen. The tibial tunnel was created with a 2.4-mm-diameter Kirschner wire from the anterior tibial cortex, with the aid of the anterior cruciate ligament tibial guide (Acufex, Smith and Nephew, Andover, MA, USA), the tip of which was placed at the native posterior medial meniscus root footprint. After finalizing the tibial tunnel position, the 18G spinal needle with loop No. 1 PDS (Ethicon, NJ, USA) was used to shuttle the free ends of the number 2 Hi-Fi suture from the articular end next to the PMMR insertion into the anterior tibial cortex. The PMMRT was reduced and stabilized by pulling both suture ends through the tibial tunnel and tied with an XO button (ConMed-Linvatec, Largo, FL, USA) at the anterior tibial cortex using five surgical square knots (Fig. 2).

### All-suture anchor repair technique

**Fig. 3:**
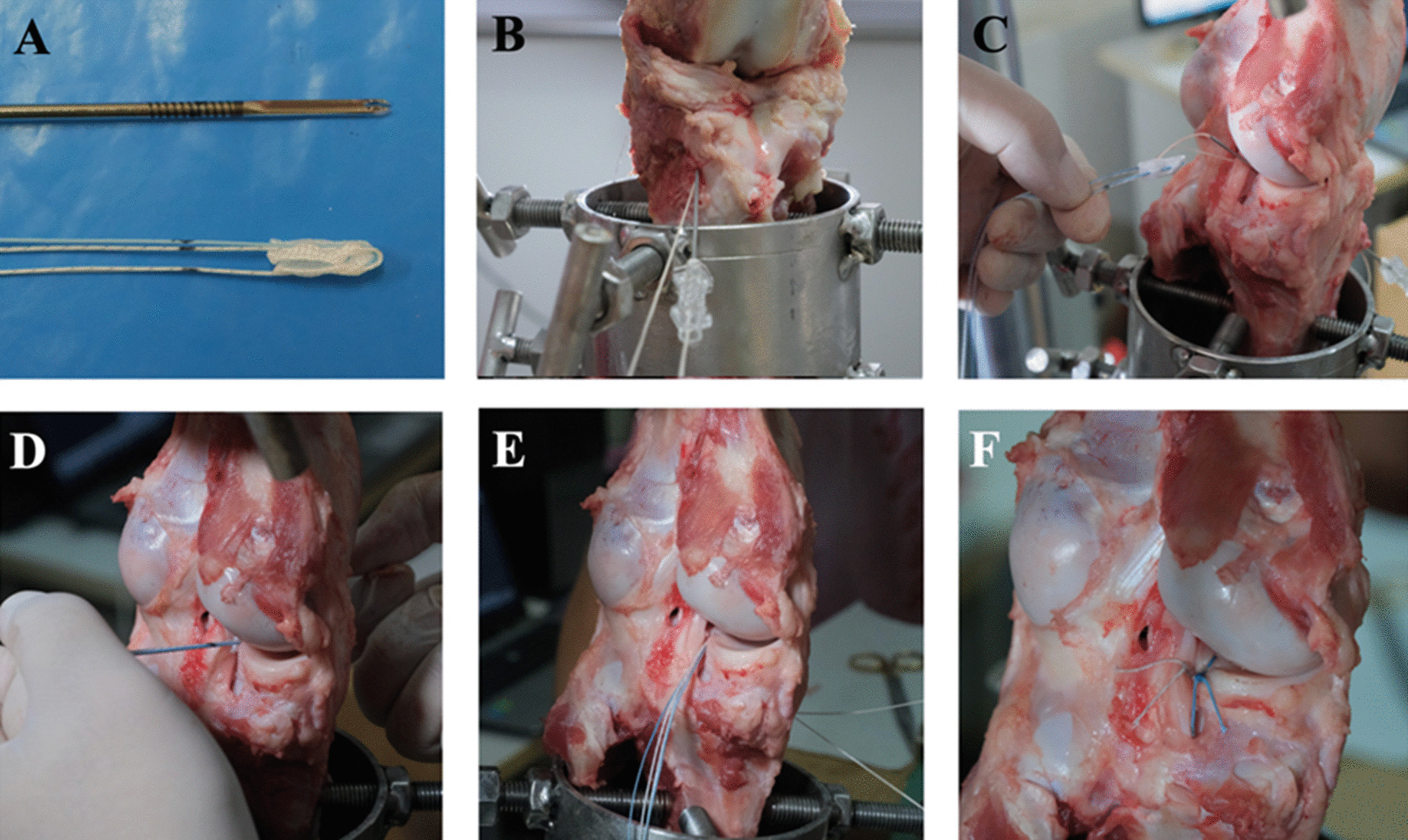
Steps for PMMRT repair with all-suture anchor repair technique. **A** The 2.8-mm Y-Knot RC anchor was unloaded from the handle and marked at 1.5 cm with the marking pen. **B** An 18G spinal needle with a looped PDS suture was inserted through the tibial tunnel in the same way as the transtibial pullout technique. **C** Unloaded Y-Knot was looped with the shuttle suture and its kink point, then pulled into the tibial tunnel. **D** The all-suture was pulled until reaching the desired marked length and then pulled backward to create the perfect deployment. **E** The four limbs of the all-suture anchor were retrieved. **F** Modified Mason–Allen suture was performed, and the final construct was illustrated

The ASA repair technique for PMMRT was modified based on an original technique described by Balke et al. [22]. In our study, we used the 2.8-mm Y-Knot RC anchors (ConMed-Linvatec Largo, FL). The anchor was marked 1.5 cm from the tip to ensure the proper depth was used for the suture ball deployment. The tibial tunnel was created with a 2.4-mm-diameter Kirschner wire from the anterior tibial cortex with the ACL tibial guide in the same fashion as TPO technique. The anchor was detached from its handle instrument. Next, the 18G spinal needle with looped PDS was used to shuttle the unloaded all-suture anchor into the tibial tunnel at its kink point. Then, the all-suture anchor was pulled downward into the bone tunnel until reaching the 1.5-cm mark and pulled backward with the aid of knot pusher to ensure a streamlined orientation. Finally, the suture ball was deployed and stuck underneath the cortex of the tibial plateau. A modified Mason–Allen suture configuration was performed using the FirstPass Mini Suture Passer (Smith and Nephew, Andover, MA, USA) at 3 mm medially to the torn edge of the posterior horn of the medial meniscus. The posterior medial meniscal root was then reattached to its footprint with five surgical square knots (Fig. 3).

### Biomechanical testing

**Fig. 4:**
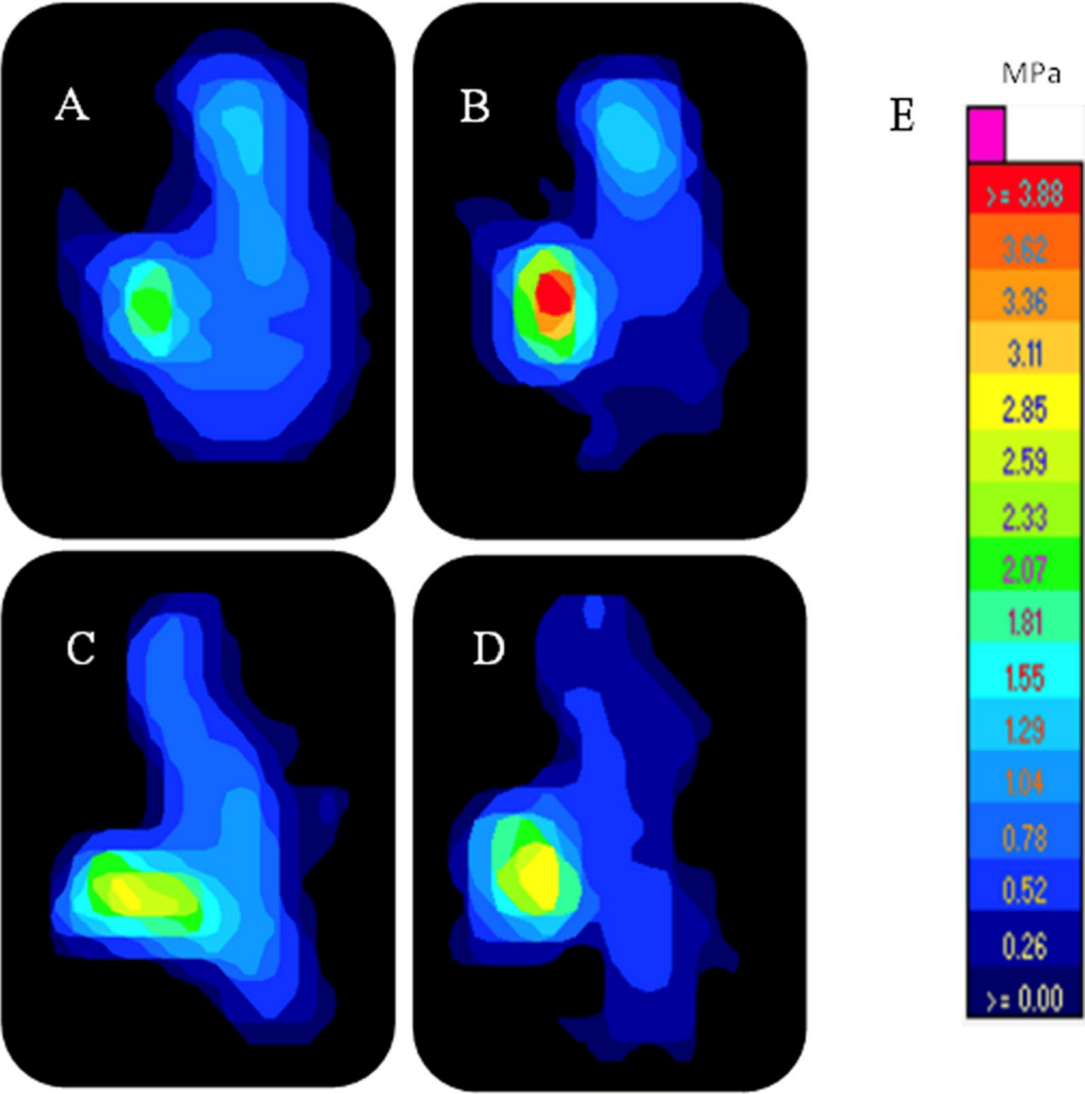
Representative medial compartment pressure map of the four testing conditions at 0° knee flexion angle. These pressure maps illustrate the distribution of contact surface area, contact pressure, and peak pressure at 0° knee flexion in different testing conditions. **A** Intact, **B** PMMRT, **C** TPO repair technique, **D** ASA repair technique, **E** Calibrated pressure; MPa, higher pressures are indicated by colors orange and yellow, and lower pressures by colors green and blue

**Fig. 5:**
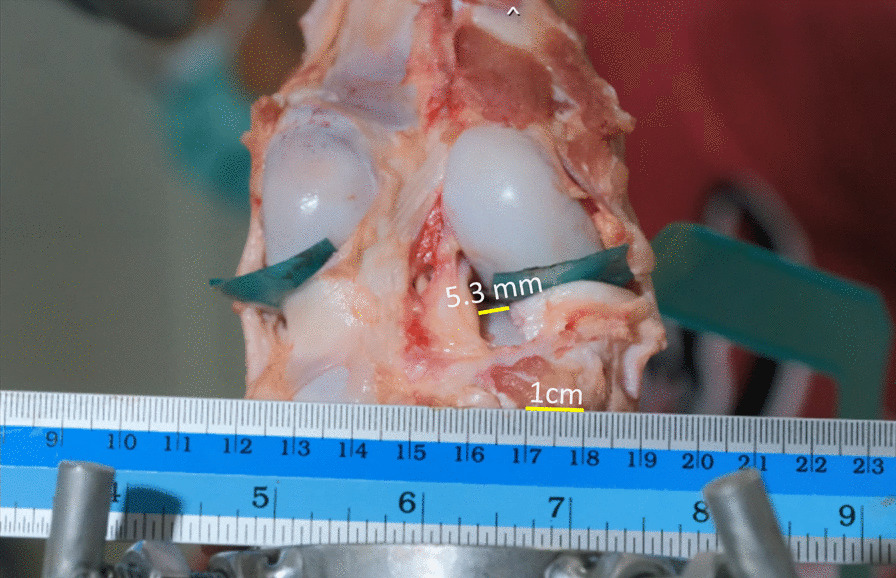
The displacement of the posterior medial meniscal root at time-zero was assessed using a calibrated ruler and subsequently recorded with a digital camera to facilitate later measurements. All captured images were uploaded to the Synapse PACS software for centralized storage and analysis. Prior to measurement, the ruler's scale was calibrated to ensure a precise measurement scale of 10 mm within the PACS system. The meniscal displacement was then determined by identifying and marking the distance between the gaps at the uppermost portion of the meniscus

A dynamic testing machine (Instron ElectroPuls E10000; Instron Systems, Norwood, MA, USA) with the ability to control the axes of the coronal and vertical motions was used. Restricting valgus–varus alignment in the coronal plane ensured an equivalent amount of distributed load on both knee compartments. The femur and tibia were secured with the custom jig rigidly attached to the base of a dynamic testing machine. The tibia baseplate was allowed for a minimal axial rotation to ensure the tibiofemoral conformity self-adjusted during the load. After certification of the final fixation in the specimens, the testing condition was conducted under a restricted position, except for the sagittal plane motion (flexion/extension). The I-scan knee pressure sensor model 4000 was inserted into the medial and lateral compartments of the joint space (Fig. 1). All knee specimens were tested by being loaded with 600 N axial compressive force at three different flexion angles (0°, 45°, and 90°), which created a contact pressure mapping by each cell of the sensor. The mean contact area, contact pressure, and peak pressure were obtained for each testing condition. The peak contact pressure was determined at the location corresponding to the peak force as automatically captured by the pressure mapping sensor. This representation is visually conveyed through colors, specifically orange and yellow hues (Fig. 4). During maximum compressive force at each flexion angle, the time-zero displacement of the meniscal root was measured with the calibrated ruler and captured by digital camera for later measurement. All pictures were uploaded in Synapse PACS software (Fig. 5). The displacement was measured and reported as an average from two assessors, and all tests were performed under room temperature. Finally, the knees were kept continuously moist with 0.9% normal saline sprinkled throughout the experiment testing to prevent dryness and reduce the additional shear force on the sensor surface.

### Pressure sensor preparation

Before the simulation with the knee specimens in the testing conditions, the I-scan knee pressure sensor model 4000 (Tekscan, Norwood, MA, USA) was inserted into the medial and lateral compartment of the joint space (Fig. 1). Unlike the previous biomechanics study [23], a submeniscal incision through the menisco-tibial portion was not required in the current study. We instead aimed to place the sensor above the meniscus to assess the tibiofemoral contact mechanics to avoid consequences from disrupting the menisco-tibial attachment, which potentially interfered with the tibiofemoral contact mechanics. Before conducting each testing condition, the pressure sensor was carefully positioned on a flat surface and subjected to a meticulous calibration process to establish a reliable baseline reference of "0." All sensors were activated during this calibration procedure to ensure their readiness for data collection. The preparation and calibration of the pressure sensors were carried out in strict accordance with the manufacturer's reference guideline [28]. Subsequently, the sensor was positioned within the knee joint for the experimental assessments. To circumvent any potential confounding effects originating from the sensor's thickness prior the application of experimental compressive force, pretensioning process was implemented and a real "0" setting was confirmed. To further ensure accuracy, an initial visualization of the signal on the sensor mapping was facilitated by applying a preload of 25 N of compressive force and recheck with the real-time data output from sensor [24]. After the sensor placement was finalized to ensure all tibiofemoral contact areas were covered, the sensor was sutured to the knee joint soft tissue to secure sensor position to prevent additional motion during testing. The pressure sensor generated a pressure map that illustrated the distribution of contact surface area, contact pressure, and peak pressures for each condition. Five seconds after reaching a stable measurement, data collection was performed. Only data from the medial compartment were selected for final analysis (Fig. 4).

### Statistical analysis

The sample size was determined by referencing the power analysis of prior analog biomechanical meniscal root studies [23, 24]. A priori power analysis was carried out using G*Power 3.1.9.7 software (Franz Paul, Kiel, Germany). With a significance level of 0.05, the analysis indicated that four samples per group would be required to achieve an effect size of *d* = 2 in the tibiofemoral contact mechanics with a power of 0.80. We further added one extra sample per group to expand the volume of available data for analysis.

Statistical analyses were carried out using Stata (version 17, College Station, TX; StataCorp LLC). Descriptive statistical analysis, represented as means with standard deviations, was used to describe parameter outcomes (contact area, contact pressure, and peak pressure). Normal distribution of the data was accessed with the Shapiro–Wilk test. To compare the difference between groups, one-way analysis of variance (ANOVA) was used for all comparisons, and the Mann–Whitney U test was used for the nonparametric comparison of two groups in the same flexion angle. In all instances, a *p* value of ≤ 0.05 was considered statistically significant**.**

## Results

### Contact surface area

**Fig. 6:**
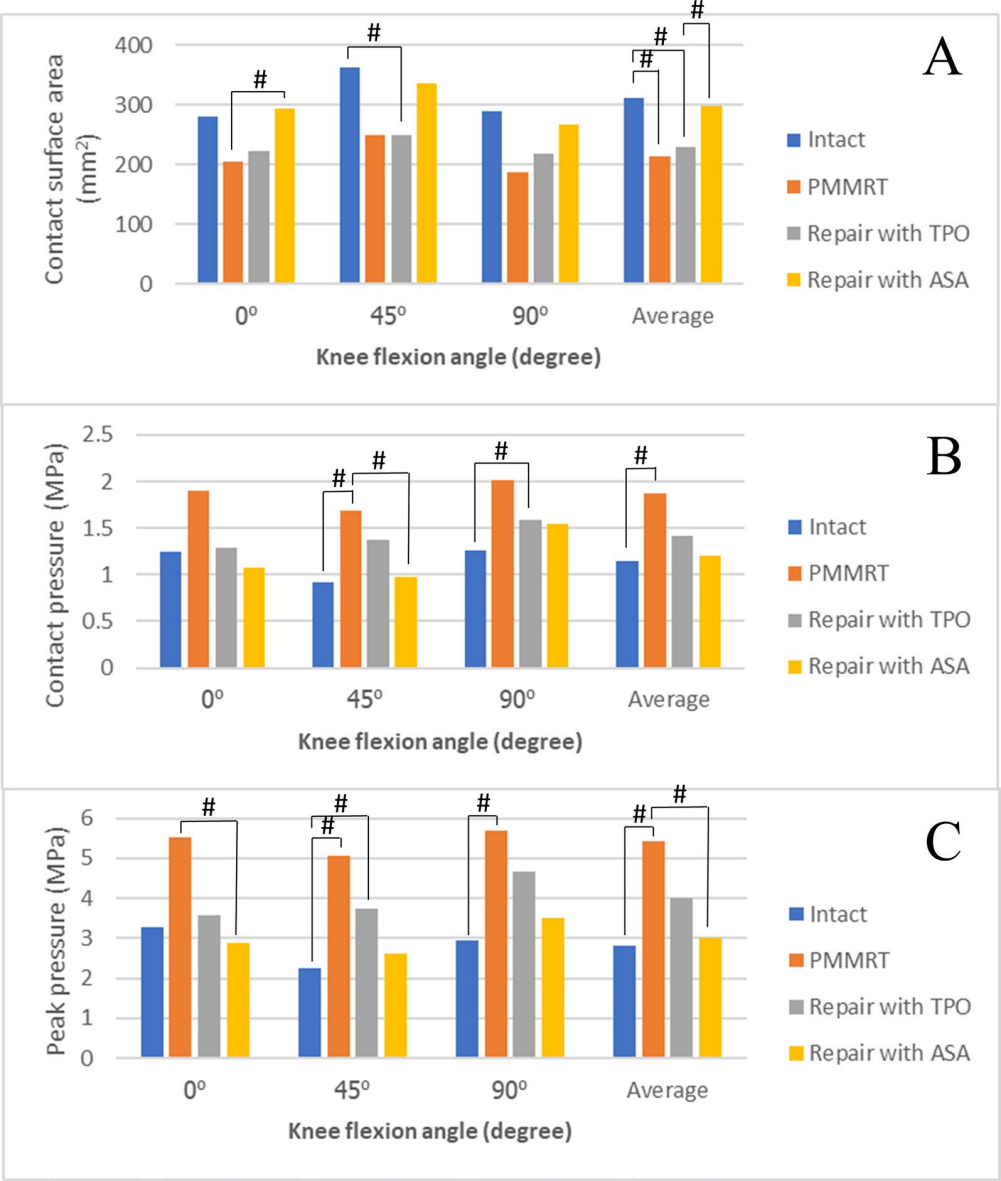
Meniscus condition effect on **A** contact surface area, **B** contact pressure, and **C** peak pressure in the medial compartment. PMMRT, posterior medial meniscus root tear; TPO, transtibial pullout technique; ASA, all-suture anchor technique.** #** Significant at level ≤ 0.05

The contact surface area of the medial compartment for each condition and knee flexion angle is presented in Fig. 6A. At each knee flexion angle, the PMMRT group demonstrated a lower contact surface area than the intact group (Table 1). When averaged across all knee flexion angles, the statistically significant reduction in contact surface area of PMRRT was 31% when compared with an intact group (*P* = 0.05; Table 2). After fixation, no significant difference existed between the ASA and intact groups, while significant differences were observed between TPO and intact groups at 45° and an average value (*P* = 0.021 and 0.016, respectively). In the comparison between two fixation techniques, the ASA group demonstrated a higher contact surface area than the TPO group at the average knee flexion angle (*P* = 0.05).

**Table 1:** Results of mean contact surface area, contact pressure, and peak pressure for each meniscal testing conditions at various knee flexion angles

Parameters	Flexion angle	Intact	PMMRT	Repair with TPO	Repair with ASA
Contact surface area (mm^2^)^∞^	0°	280 ± 67.48	204.8 ± 59.43	222.8 ± 63.66	293.6 ± 47.53
	45°	363.8 ± 75.25	248.2 ± 129.77	248.2 ± 58.20	337 ± 69.96
	90°	290 ± 57.92	187.2 ± 59.89	218.8 ± 39.48	267.20 ± 101
	Average	311.27 ± 73.31	213.4 ± 86.89	229.93 ± 52.46	299.27 ± 76.84
Contact pressure (MPa)^∞^	0°	1.24 ± 0.21	1.9 ± 1.21	1.29 ± 0.33	1.07 ± 1.07
	45°	0.92 ± 0.16	1.69 ± 0.79	1.38 ± 0.36	0.98 ± 0.22
	90°	1.26 ± 0.41	2.02 ± 0.50	1.59 ± 0.50	1.54 ± 0.56
	Average	1.14 ± 0.31	1.87 ± 0.83	1.42 ± 0.40	1.2 ± 0.42
Peak pressure (MPa)^∞^	0°	3.29 ± 1.18	5.53 ± 2.22	3.57 ± 1.66	2.88 ± 1.24
	45°	2.26 ± 0.54	5.05 ± 1.98	3.75 ± 0.83	2.63 ± 1.17
	90°	2.94 ± 1.02	5.7 ± 1.62	4.66 ± 2.24	3.52 ± 1.25
	Average	2.83 ± 0.99	5.43 ± 1.83	3.99 ± 1.63	3.01 ± 1.2

*PMMRT* Posterior medial meniscus root tear, *TPO* Transtibial pullout technique, *ASA* All-suture anchor technique

^∞^Value presented as mean ± standard deviation

**Table 2:** Comparison of mean contact surface area, contact pressure, and peak pressure between meniscal testing conditions at various knee flexion angles

Parameters	Flexion angle	All condition^∞^	Intact versus PMMRT^µ^	Intact versus repair with TPO^µ^	Intact versus repair with ASA^µ^	PMMRT versus repair with TPO^µ^	PMMRT versus repair with ASA^µ^	Repair with TPO versus repair with ASA^µ^
Contact surface area (mm^2^)	0°	0.89	0.095	0.463	0.528	0.69	0.036*	0.207
	45°	0.23	0.151	0.021*	0.841	0.834	0.222	0.094
	90°	0.087	0.056	0.056	0.841	0.209	0.151	0.69
	Average	0.042*	0.05*	0.016*	0.841	0.841	0.095	0.05*
Contact pressure (MPa)	0°	0.112	0.421	0.548	0.222	0.69	0.059	0.31
	45°	0.052	0.021*	0.059	0.834	0.548	0.032*	0.173
	90°	0.012*	0.056	0.032*	0.421	0.209	0.31	0.834
	Average	0.071	0.016**	0.095	0.753	0.209	0.056	0.209
Peak pressure (MPa)	0°	0.118	0.095	0.841	0.841	0.116	0.032*	0.69
	45°	0.155	0.016*	0.032*	0.421	0.31	0.056	0.095
	90°	0.065	0.016*	0.222	0.421	0.421	0.056	0.421
	Average	0.006**	0.008**	0.058	0.548	0.151	0.008**	0.222

*PMMRT* Posterior medial meniscus root tear, *TPO* Transtibial pullout technique, *ASA* All-suture anchor technique

^∞^The result are shown as *P* value analyzed by the one-way ANOVA test

^µ^The result are shown as *P* value analyzed by the Mann–Whiney U test

*Significant at level ≤ 0.05

**Significant at level ≤ 0.01

### Contact pressure

The contact pressure of medial compartment for each condition and knee flexion angle is presented in Fig. 6B. At each knee flexion angle, the PMMRT group demonstrated higher contact pressure than the intact group, with a significant difference at 45° knee flexion (Tables 1, 2). When averaged across all knee flexion angles, the statistically significant increase in contact pressure of PMRRT was 64% when compared with an intact group (*P* = 0.016; Table 2). After fixation, no significant difference existed between the ASA and intact group, while significant differences were observed between the TPO and intact groups at 90° value (*P* = 0.032). In the comparison between fixation techniques, no significant difference existed in the contact pressure observed between both groups.

### Peak pressure

The peak pressure of the medial compartment for each condition and knee flexion angle is presented in Fig. 6C. At each knee flexion angle, the PMMRT group demonstrated higher peak pressure than the intact group (Table 1). When averaged across all knee flexion angles, the statistically significant increase in peak pressure of the PMMRT was 91% when compared with an intact group (*P* = 0.008; Table 2). After fixation, no significant difference existed between the ASA and intact group, while significant differences were observed between the TPO and intact groups at 45° value (*P* = 0.032). In the comparison between the fixation techniques, no significant differences existed in the peak pressure observed between both groups.

### Time-zero displacement

**Fig. 7:**
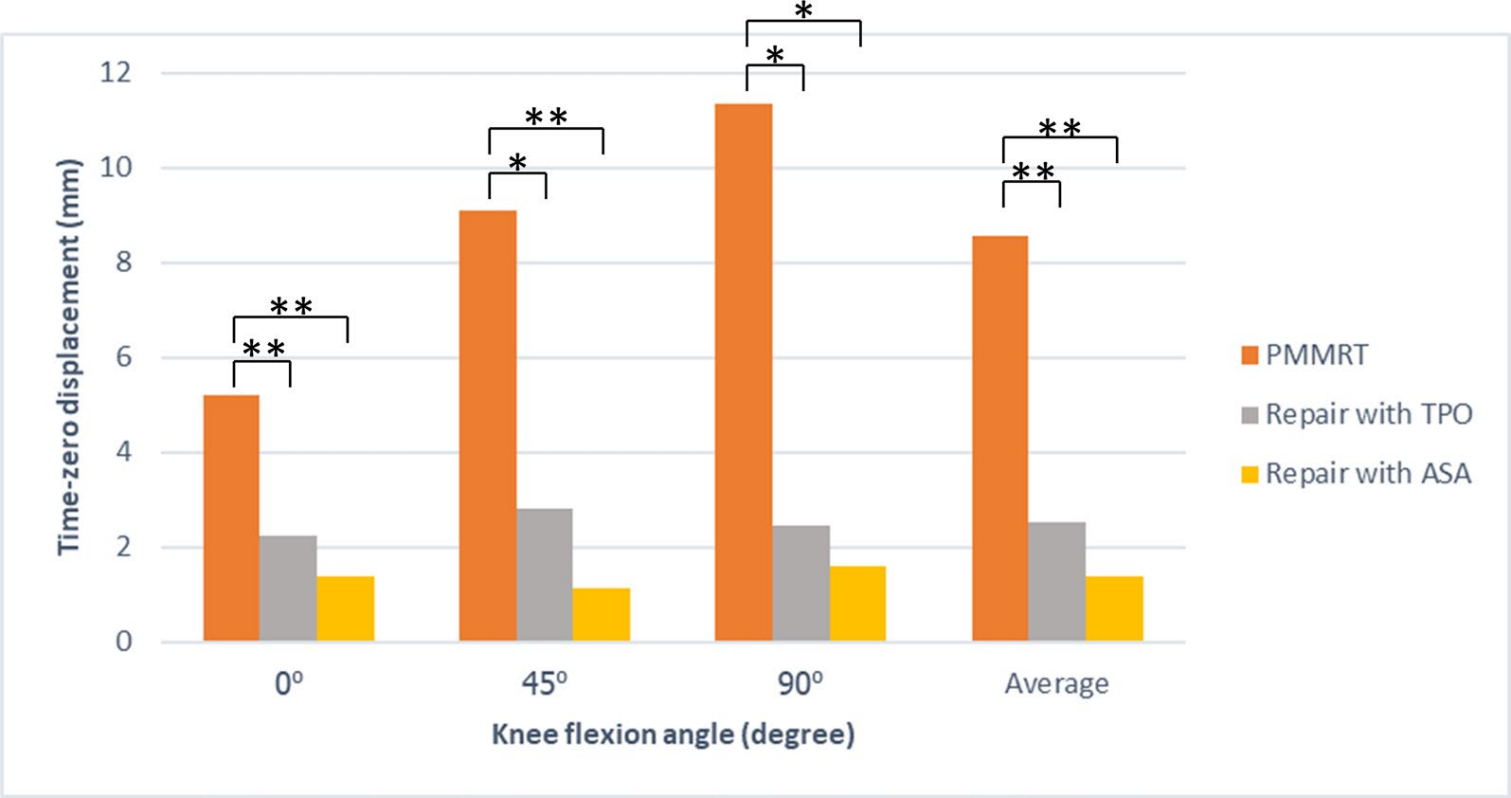
The bar graphs demonstrate the time-zero displacement of the posterior medial meniscus root by comparing between the PMMRT condition, TPO repair technique for PMMRT, and ASA repair techniques for PMMRT. PMMRT, posterior medial meniscus root tear; TPO, transtibial pullout technique; ASA, all-suture anchor technique. *Significant at level ≤ 0.05, **Significant at level ≤ 0.01

The mean time-zero displacement of the PMMRT increased with a higher degree of knee flexion. Both repair techniques had the potential to reduce the tear displacement but could not restore to zero as an intact condition. The TPO group reduced time-zero displacement when compared with the PMMRT group with a statistically significant difference at all flexion angles (*P *< 0.05). In the same fashion, the ASA group also reduced time-zero displacement when compared with the PMMRT group with a statistically significant difference at all flexion angles (*P *< 0.05). The ASA group tended to have lower displacement compared to the TPO group. However, the difference was not achieved at a statistically significant level (Fig. 7).

## Discussion

The primary objective of this study was to compare the biomechanical properties of TPO and ASA techniques for PMMRT repairs. As hypothesized, the most significant finding was that the ASA technique demonstrated superior biomechanical properties in terms of contact surface area, as compared with the TPO technique. Moreover, the ASA technique could restore the same tibiofemoral contact mechanics as the native knee, without any statistically significant difference between the groups.

This study showed that PMMRT affected the tibiofemoral mechanics by significantly decreasing the contact surface area and increased contact pressure and peak pressure based on the reference values observed in the intact meniscus group. These findings mostly concurred with prior studies that evaluated how PMMRT affected biomechanical properties. For instance, Chung et al. [29] investigated the effect of the fixation technique for PMMRT in a porcine model and reported that the technique resulted in a significant increase in peak contact pressure (ranging from 52 to 73%) and decreased the contact area (ranging from 52 to 55%). Saltzman et al. [30] evaluated the biomechanical effect of PMMRT in human fresh-frozen cadavers. Across varied knee flexion angles, they showed an overall decrease in contact surface area (26%) and an increase in contact pressure and peak pressure (24% and 10.6%, respectively) with PMMRT compared to the intact condition. Despite the different models used, the consistent trend of tibiofemoral mechanics remained evident when comparing our results with previous analogous studies. These similar findings support the competence of our study protocol in representing the condition regarding the biomechanical effect of PMMRT.

The assessment of individual flexion angles in our study is summarized by presenting the average value across all measured angles. This methodology aligns with the approach of previous studies [23, 30], which emphasized the significance of reporting an averaged value encompassing all flexion angles as a notable outcome. The rationale behind this approach is to succinctly capture the consistent findings observed across the various flexion angles assessed. Consequently, we deemed it judicious to adopt a similar presentation method to enhance comparability and foster a comprehension of the results.

Apart from the biological aspect, the optimal biomechanical properties of the fixation technique are important for the meniscal healing process. Generally, the significant clinical improvement has been shown to occur at 1–3-years postoperative follow-up after using the TPO technique [19, 31–33]. Nevertheless, the controversial results following the use of the TPO technique have been reported by Level 3–4 clinical outcome studies [19, 32, 33]. Previous studies, when compared to suture anchor repair technique, revealed less satisfactory postoperative outcomes regarding the effect of meniscal extrusion [19, 33] and incomplete healing of the PMMRT based on second-look arthroscopic findings [32]. Theoretically, the TPO technique has several drawbacks. In the concept of ACL reconstruction surgery, distal fixation of the graft has been related to diminished biological incorporation of the graft healing due to sagittal and longitudinal graft–tunnel micromotion [34]. Likewise, the distal fixation of the PMMRT with the long meniscal-suture construct via the TPO technique potentially impaired healing of the meniscal root due to additional micromotion and a reduction in stiffness of the meniscal root repair complex under the maximal compressive load [17]. The longitudinal motion of the suture within the tibial bone tunnel could also lead to tunnel widening prior to complete meniscal healing. Meanwhile, adequate suture tensioning is a challenge due to the long distance between the meniscal root and fixation point [19]. Kim et al. investigated the radiological and clinical outcomes following PMMRT repair using the TPO and suture anchor techniques. The researchers found no significant difference in clinical outcomes between both groups at a 26-month follow-up. However, the TPO technique, based on MRI results, demonstrated a higher incidence of incomplete healing than the suture anchor techniques [19].

Only a small number of studies have investigated the biomechanical properties of the suture anchor technique for PMMRT repairs [16, 35]. In a porcine biomechanical study, Feucht et al. [16] found that, compared with the TPO technique, the suture anchor technique showed statistically significant superior biomechanical properties in terms of displacement after cyclic load, stiffness, and maximum load to failure, as compared with the TPO technique. Wu et al. [35] also conducted a study on the porcine model and, correspondingly, reported lower displacement during cyclic loading with the suture anchor technique compared to the TPO technique. PMMRT repairs done with an all-suture anchor are claimed to offer an advantage compared with a conventional suture anchor, but no prior biomechanical-based proof exists to confirm the claim [22] since a discrepancy in biomechanical performance might exist between different types of suture anchors [36, 37]. Barber et al. evaluated the biomechanical properties of all-suture anchors (2.8-mm Y-Knot) in porcine cortical bone and polyurethane block compared with native PMMR. The researchers reported that all-suture anchor constructs provided the maximal ultimate failure strength equal to tenfold that of the native PMMR (602.9 ± 159 N versus 61.1 ± 20.2 N, respectively). Less than 50% of broken sutures were found with all-suture anchors when compared with other suture materials, and the anchors could maintain the insertion without further displacement [38]. Our superior biomechanical result from the ASA technique has the same outcome as achieved in previous suture anchor studies. Based on our findings, then, the ASA technique might be favorable over the TPO technique. However, long-term clinical studies are required to prove this potential benefit of the ASA technique.

Regarding the time-zero displacement, the results showed that both repair techniques could significantly decrease the displacement compared with the PMMRT; however, no such technique could restore to zero as an intact condition. As demonstrated in a previous biomechanical model, using a non-anatomical attachment of the PMMR of only 3–5 mm significantly compromised meniscal function [23, 39]. Starke et al. [39] reattached the medial meniscal root at 3 mm more medial than the native footprint, which significantly reduced the hoop stress tension and resulted in cartilage deformation under the tibiofemoral compressive load. Based on our findings, the PMMRT was increased beyond the 3-mm limit at every degree of knee flexion. After repair, none of the techniques revealed a higher value exceeding this limit.

It is important to note that our studies incorporated an axial load that closely mimics the full weight of physiological load. In clinical practice, patients would advise to engage in partial weight bearing during the acute postoperative period, resulting in loading values lower than those used in our experiments. Fukubayashi et al. conducted a study indicating that, at a lower load of 200 N in their experimental setup, the contact area primarily resided within the meniscus itself, accounting for up to 72% of the total contact area. Conversely, as the load was increased, the percentage of contact area attributable to the meniscus exhibited a decline [40]. This disparity may potentially impact the significance of our results as well. However, it is crucial to acknowledge that partial weight bearing is a subjective matter and can vary significantly among individuals. Eickhoff et al. [41] has shown that many patients struggle to adhere to loading limitations even a few days after surgery, even with the guidance of a physiotherapist, often leading to excessive weight bearing. Given these considerations, we believe that setting the axial load at a higher value, closer to full physiological weight, may prove more beneficial than using a lower value, as the latter might not accurately reflect real-life scenarios.

This study has several limitations. First, the porcine knee model cannot represent the identical consequence of the PMMRT and after these repair techniques in the human knee. Nevertheless, the porcine knee model has been considered a valid animal knee model and remains widely used in the orthopedic field, especially for meniscal root studies, due to the analogous function between the model and the anatomy of the human knee with persistent material properties [16, 29, 35, 38]. Thus, the finding regarding the relative biomechanical performance of the repair technique is unlikely to have been biased by the application of the porcine knee model. Second, due to deriving from an in vitro controlled experimental study, these biomechanical results do not include the determination of the biological factor effect; for example, the hoop stress effect, meniscal healing, and cartilage status are not included. Moreover, the physiological loading of the meniscus usually involves a combination of shearing and compressive force [42], a combination not represented by our study protocol. However, our protocol was set up the same way as many prior meniscal root biomechanical studies that apply a simplification of complex biomechanical properties with in vivo knee joints [7, 23, 29, 30]. Third, our biomechanical model, while valuable for our study, serves as a simplified representation of the intricate in vivo conditions encountered by the knee joint during functional activities. It is important to acknowledge that physiological meniscal loading encompasses both static and dynamic patterns, which are not fully captured in our testing model. Despite this limitation, our setup offered a reproducible and consistent loading scheme, enabling reliable comparisons between different conditions. Moreover, the utilization of this standardized approach has been widely adopted in several similar studies [5, 14, 23, 29], facilitating a more direct comparison to the existing body of literature. Fourth, only the modified Mason–Allen suture configuration was used for both repair techniques in our study. This suture configuration has been reported to provide superior tibiofemoral contact mechanics compared to different types of constructs [28]. Thus, the results from the current study cannot be widely generalized for other suture materials or different suturing techniques.

## Conclusion

The ASA technique demonstrated superior biomechanical properties in terms of contact surface area, as compared with the TPO technique under compressive loading conditions. The ASA technique could also restore the tibiofemoral contact mechanics compared with the native intact knee. Meanwhile, the significant difference in tibiofemoral mechanics compared with the intact knee could be observed in the TPO technique. When addressing concerns regarding biomechanical properties following PMMRT repair, the implementation of the ASA technique appears to offer potential advantages in restoring joint contact mechanics as compared to the native knee. Additionally, it is imperative to recognize that both repair techniques exhibit a time-zero displacement inferior to that of the intact knee. Therefore, a prudent approach entails advocating for a gradual rehabilitation protocol following PMMRT repair.

## Data Availability

The datasets generated and/or analyzed during the current study are available from the corresponding author upon any reasonable request.

## Abbreviations

TPO: Transtibial pullout
ASA: All-suture anchor
PMMRT: Posterior medial meniscal root tear
MRI: Magnetic resonance imaging
